# Age related changes in microglial phenotype vary between CNS regions: Grey versus white matter differences

**DOI:** 10.1016/j.bbi.2011.11.006

**Published:** 2012-07

**Authors:** Adam D. Hart, Andreas Wyttenbach, V. Hugh Perry, Jessica L. Teeling

**Affiliations:** Centre for Biological Sciences, University of Southampton, Southampton General Hospital, Southampton, UK

**Keywords:** Ageing, Microglia, Regional differences, Behaviour, LPS, CD11c, White matter

## Abstract

Subtle regional differences in microglial phenotype exist in the adult mouse brain. We investigated whether these differences were amplified during ageing and following systemic challenge with lipopolysaccharide (LPS). We studied microglial morphology and phenotype in young (4mo) and aged (21mo) C57/BL6 mice using immunohistochemistry and quantified the expression levels of surface molecules on microglia in white and grey matter along the rostral-caudal neuraxis. We detected significant regional, age dependent differences in microglial phenotypes, with the microglia of white matter and caudal areas of the CNS exhibiting greater upregulation of CD11b, CD68, CD11c, F4/80 and FcγRI than grey matter and rostral CNS areas. Upregulation of CD11c with age was restricted to the white matter, as was the appearance of multinucleated giant cells. Systemic LPS caused a subtle upregulation of FcγRI after 24 h, but the other markers examined were not affected. Burrowing behaviour and static rod assays were used to assess hippocampal and cerebellar integrity. Aged mice exhibited exaggerated and prolonged burrowing deficits following systemic LPS injection, while in the absence of an inflammatory challenge aged mice performed significantly worse than young mice in the static rod test. Taken together, these findings show that the effects of age on microglial phenotype and functional integrity vary significantly between CNS compartments, as do, albeit to a lesser extent, the effects of systemic LPS.

## Highlight

► This work describes age related changes in microglial phenotype in different CNS regions and highlights differences between grey and white matter.

## 1 Introduction

Microglia are the resident immunocompetent and phagocytic cells in the CNS that play a critical role in normal functioning of the CNS. They respond to injury, damage and pathogens by rapidly changing their phenotype and secretion of a plethora of soluble factors. The microglia also play a key role in the communication of systemic infection and inflammation to the brain resulting in behavioural changes, but this signalling is not detrimental to the adult healthy brain and rather contributes to recovery and maintenance of homeostasis (Dantzer and Kelley, 2007; Teeling and Perry, 2009). Microglia can become activated or ‘primed’ in chronic neurodegenerative or inflammatory diseases, and these primed cells, in contrast to the normal resident microglia, have a lower threshold for activation and can become harmful upon further stimulation (Cunningham et al., 2009; Perry et al., 2010). The normal ageing process can also induce microglia priming (Chen et al., 2008; Frank et al., 2010; Godbout et al., 2005) but the mechanism underlying these age-related changes in microglial cells are not understood. This study aimed to investigate if the age-related changes in microglia phenotype show regional differences and whether these are associated with functional changes or previously described age-related changes in neuronal integrity.

Microglial cells are long lived, myeloid-derived cells that populate the CNS during early development (Alliot et al., 1999; Ginhoux et al., 2010; Lawson et al., 1992). It is estimated that the adult mouse brain contains approximately 3.5 million microglia (Lawson et al., 1990; Long et al., 1998). Their morphology and density, however, is region specific and can range from 5% up to 12% of total cells per region, with higher densities found in the grey matter (Lawson et al., 1990). The distribution and phenotype of microglia also differs between regions in the human brain but, in contrast to rodents, the white matter contains significantly more microglia, about 13% of total glial cell density, with grey matter showing a low microglial density (Mittelbronn et al., 2001).

To gain more insight into the cellular functions of microglia in the adult mouse brain, De Haas et al. (2008) compared the cellular expression level of a number of functional surface molecules in different brain regions and found distinct regional differences. For example, the expression levels of CD11b and CD40 in the cerebral cortex were significantly lower than the levels in the spinal cord. The different regional expression of some immune molecules on microglia may reflect different aspects of microglial activation, which is of interest in the context of the rostro-caudal gradient of reactivity to injury and inflammatory stimuli in the CNS. Lesions to spinal cord promote more extensive leucocyte recruitment and blood–brain barrier breakdown than comparable lesions to cortex (Schnell et al., 1999a). The rostro-caudal gradient is also observed following focal cytokine injections with more overt leucocyte recruitment in the caudal than forebrain regions (Phillips and Lampson, 1999; Phillips et al., 1999; Schnell et al., 1999b).

With age the distribution and number of microglia changes little, if at all (Deng et al., 2006; Long et al., 1998; Ogura et al., 1994). In contrast, age-related changes in phenotype and functional properties of microglial cells have been widely reported. In the healthy adult brain, microglia display a down-regulated phenotype characterized by low expression of functionally relevant molecules such as CD45, CD68 and MHC class II (Aloisi, 2001; Perry et al., 2007) and a low phagocytic activity, but the expression levels of these molecules increase after acute CNS injury or ageing (Conde and Streit, 2006; DiPatre and Gelman, 1997; Ogura et al., 1994; Perry et al., 1993; Rogers et al., 1988; Streit, 1996). In the aged rat brain there is an increase in CD68 + cells throughout the parenchyma in both grey and white matter and appearance of MHCII positive aggregates of cells in and adjacent to white matter (Perry et al., 1993). Similar changes have been observed in aged mice. These changes have been associated with an increased sensitivity to systemic inflammatory challenge with increased cytokine production and altered behavioural responses (Barrientos et al., 2006; Chen et al., 2008; Henry et al., 2009; Wynne et al., 2010).

Many studies on age-related changes in microglia phenotype and function during ageing have focused on single regions and have not addressed possible regional differences within the CNS. Microglia activation is evident in the white matter of the cerebral hemispheres of old rats (Ogura et al., 1994), old monkeys (Sheffield and Berman, 1998; Sloane et al., 1999), and elderly humans (Simpson et al., 2007), and it has been reported that the extent of microglial cell activation in white matter, as measured by increased expression of MHCII and iNOS, is related to the degree of cognitive impairment (Sloane et al., 1999). The aim of this study was to compare the phenotype and morphology of microglia in various regions of young (4 months) and aged (21 months) mouse brain using a range of functional surface markers and to assess their phenotype following a systemic inflammatory challenge. We selected eight distinct regions of grey or white matter distributed along a rostral-caudal neuraxis. The regions included in our study were: striatum, corpus callosum, fimbria, dentate gyrus, substantia nigra, cerebellar nuclei, molecular layer of the cerebellar cortex and the inferior cerebellar peduncle. The striatum is a mixed white/grey matter region – we studied the most caudal segment of the putamen, an area that is mostly grey matter. The corpus callosum and fimbria are rostral white matter areas, while the dentate gyrus is a grey matter region from the hippocampus. The substantia nigra is a grey matter area caudal to the hippocampus with a particularly high microglial density (Lawson et al., 1990). Within the cerebellum we analysed the white matter tracts of the inferior cerebellar peduncle, the deep cerebellar nuclei, which represent a mixture of white and grey matter, and the molecular layer, which is grey matter neuropil of the cerebellar cortex.

The functional markers used in this study were selected for their sensitivity to changes in the activation state of microglia and their relevance to microglial function. CD11b and CD11c are adhesion molecules that play a role in cell migration and phagocytosis, CD68 is involved in phagocytosis and MHCII is important for antigen presentation (Kettenmann et al., 2011). FcγRs bind IgG, and play a role in antigen presentation and uptake of opsonised cell debris (Nimmerjahn and Ravetch, 2008). F4/80 contributes to peripheral tolerance induction in T regulatory cells by myeloid cells (Lin et al., 2005), Dectin-1 is a non-TLR pattern recognition receptor expressed during alternative activation of macrophages (Shah et al., 2008) and DEC-205 is a dendritic cell marker involved in antigen presentation (Jiang et al., 1995). These markers are myeloid cellspecific within the CNS and up-regulated upon cell activation (Buttini et al., 1996; Lunnon et al., 2011; Ponomarev et al., 2005; Qin et al., 2004; Shah et al., 2008). In this study we show that microglial age-related phenotypes vary regionally, with evidence of a differential expression of myeloid antigens along the rostro-caudal neuraxis. These phenotype differences correlate with age-related behavioural deficits dependent on hippocampus and cerebellum integrity.

## 2 Materials and methods

### 2.1 Animals and procedures

Female C57BL/6 mice (Harlan, UK, bred in house) were used in all experiments. Mice were housed in groups of 5–10 in plastic cages with sawdust bedding and standard chow diet and water available *ad libitum*. The holding room temperature was kept between 19 and 23 °C with a 12:12 h light–dark cycle (light on at 0700 h). Young mice were 4 months old and aged mice were 20–21 months old (*n* = 10–15 per treatment group). Changes in behaviour and microglial phenotype were assessed in the same cohort of mice. All procedures were performed under the authority of a UK Home Office License in accordance with the UK animals (Scientific Procedures) Act 1986, and after obtaining local ethical approval by the University of Southampton.

### 2.2 LPS administration

Mice were injected intraperitoneally with saline or LPS at a dose of 100 μg/kg (L5886, Salmonella abortus equi, Sigma, Poole, UK).

### 2.3 Burrowing

Burrowing behaviour was assessed as described previously (Teeling et al., 2007). Briefly, plastic cylinders 20 cm long and 6.8 cm in diameter and fixed at a slight incline were filled with 190 g of normal food diet pellets and placed in individual cages. Burrowing activity was measured between 3 and 5 h after saline or LPS injection by weighing the amount of displaced food pellets, after which the tube was refilled to measure overnight burrowing activity. Baseline burrowing activity over 2 h or overnight was determined for each mouse 24 h prior to the experiment to allow the expression of data as percentage of baseline activity.

### 2.4 Multiple static rod test

Static rod test performance was assessed as previously described (Contet et al., 2001) with minor adaptations. Three wooden rods of varying diameter (35, 22 and 9 mm) each 60 cm long were fixed on one end to a supporting platform and suspended 60 cm above a bed of foam. A mouse was placed at the end of the rod facing towards the open end. The time taken to orientate 180 degrees (“orientation”) and the time to travel to the wooden platform (“transit time”) were then noted. If the mouse failed to reach the wooden platform, it was assigned a score of “fail”. The multiple static rod test was performed between 1 and 2 h after saline or LPS injection. A baseline measurement was taken 24 h prior to the experiment. Prior to baseline mice were habituated to all three rods. All mice successfully traversed the two larger rods, therefore only data from the smallest rod is presented.

### 2.5 Rod climbing assay

An L-shaped metal rod of 2 mm diameter and 28 cm length was suspended from a wire mesh screen 0.5 m above a bed of foam. The mouse was placed at the bottom of the rod and allowed to climb for 60 s to reach the wire mesh screen. Mice were scored as follows: fell within 10 s (=1), 25 s (=2) or 59 s (=3), remained on the rod for > 60 s (=4), or reached the inverted screen within 60 s (=5), 25 s (=6), or 10 s (=7).

### 2.6 Tissue processing and immunohistochemistry

24 h after LPS or saline injection mice received a terminal dose of pentobarbital and, following transcardiac perfusion with heparinised saline, brain and spleen tissue were immediately removed, embedded and frozen in optimal cutting temperature (OCT) medium (Sakura Finetek, Thatcham, UK). 10 μm sections were cut on a cryostat in the coronal plane at −0.9, −3.0 or −6.0 mm ± 0.3 mm from bregma, air dried and frozen at −20 °C until required.

For immunohistochemical analysis, sections were dried at 37 °C and fixed for 10 min in 100% ethanol at 4 °C. Sections were then quenched with 3% hydrogen peroxide (Sigma, Poole, UK) in phosphate buffered saline (PBS) and blocked with appropriate 10% animal serum (Vector Laboratories, Peterborough, UK) and 1% bovine serum albumin (Fisher Scientific, Loughborough, UK). Primary antibodies were incubated overnight at 4 °C, for details see Table 1. Biotinylated secondary antibodies, either rabbit-anti-rat IgG or goat-anti-hamster IgG (Vector Laboratories, Peterborough, UK), were added for 45 min, followed by exposure to avidin biotin complexes (Vector Laboratories, Peterborough, UK) and DAB (3,3′-Diaminobenzidine) (Sigma, Poole, UK). Sections were counterstained with Harris haematoxylin (Sigma, Poole, UK). If prepared for immunofluorescence sections were incubated with a donkey-anti-rat or goat-anti-rabbit IgG secondary antibody conjugated with a 488 or 568 nm fluorophore (Invitrogen, Paisley, UK) or with biotinylated secondary antibodies followed by 488 or 568 nm fluorophore conjugated streptavidin (Invitrogen, Paisley, UK). Specificity of primary antibodies was confirmed using spleen as a positive control and omission of the primary antibody as a negative control. The specificity of FcγRI staining was confirmed using brain tissue from ME7 infected Fc gamma chain deficient mice.

### 2.7 Quantification of immunohistochemistry

Images were analysed and quantified using ImageJ. The DAB and haematoxylin channels were isolated using a plugin and a threshold was determined for quantification. Thresholds were determined for each experiment to control for variation in DAB staining intensity between experiments. Background or excessively dark haematoxylin staining was removed using the “despeckle” setting and, when required, by superimposing a mask of the haematoxylin channel onto the image. The region of interest was traced by “freehand” from the image and the average pixel density within the selected area was calculated. For each animal (*n* = 4–5 per treatment group), two images per region of interest were captured at ×20 magnification for quantification, using a brain atlas to identify matching regions of interest in each hemisphere. The average pixel density above threshold of the two images was calculated and data expressed as fold increase over 4 month old, saline treated expression levels in the same region. FcγRI expression in the striatum was excluded from analysis due to non-specific nuclear binding in this particular region.

### 2.8 Quantitative PCR

Brain tissue was rapidly removed following perfusion and dissected to separate the cerebellum from a coronal section of hippocampus, thalamus and cortex (bregma -1.5 mm to -3.5 mm). The coronal section was divided into two hemispheres, snap frozen in liquid nitrogen and stored at -80 °C. Total RNA was extracted from brain tissue using RNeasy mini kits (Qiagen, Crawley, UK) and treated with DNAse I to remove any contaminating gDNA (Qiagen). cDNA was synthesised using reverse transcription reagents from Applied Biosystems (Warrington, UK). SYBR green super mix (BioRad, Hemel Hempstead, UK) was used to detect amplification of primer products. IL-1β primers were purchased from Invitrogen and iNOS, GAPDH and IL-6 primers were purchased from Sigma, Poole, UK. Primer sequences are as previously described (Palin et al., 2008; Sato et al., 2003). Samples were quantified against a standard curve using mouse hippocampus tissue infected with ME7, injected intraperitoneally with LPS and collected 6 h after injection as a positive control. The amount of mRNA was then estimated as the ratio of GAPDH. *n* = 3–4 per treatment group.

### 2.9 Statistical analysis

Data sets were tested for a normal distribution using the D’agostino-Pearson omnibus test. All tests were performed in either Sigmaplot 11.0 or GraphPad Prism 5.0. Overnight burrowing data was normally distributed and was analysed using two way ANOVAs with Holm-Sidak post tests. Two hour burrowing data was not normally distributed and was therefore analysed using Mann–Whitney tests on saline and LPS groups. Pass/fail data from the multiple static rod tests was analysed using a Chi squared test. Transit time data was analysed using a Mann–Whitney test. Quantification of the immunohistochemical analysis was performed by expressing data as fold increase from the mean of the 4 month old saline values from the same brain region, logarithmically transformed and analysed using a three way ANOVA with Holm-Sidak post tests. Quantitative PCR data was logarithmically transformed and analysed by two way ANOVA and Holm-Sidak post tests.

## 3 Results

### 3.1 Microglial morphology changes with age

**Fig. 1:**
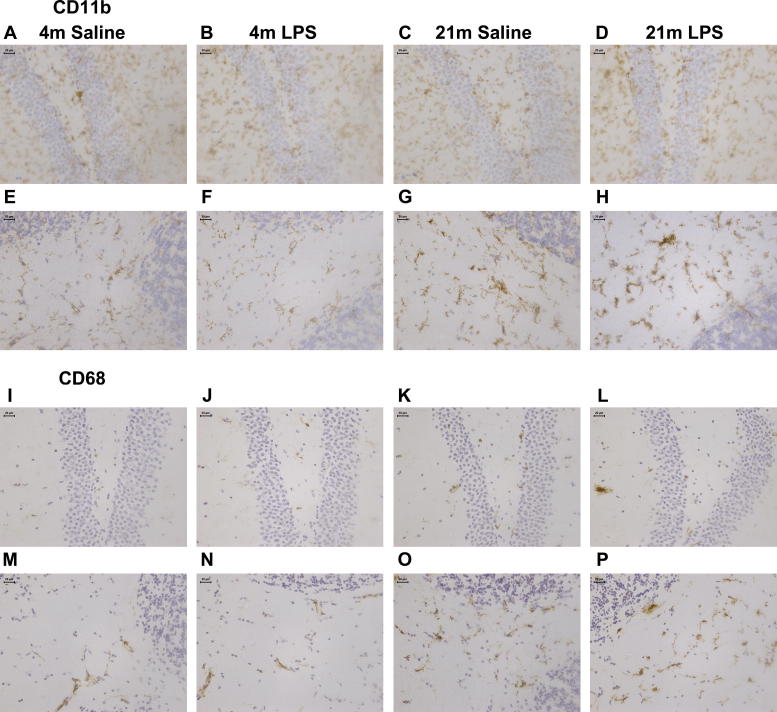
CD11b and CD68 marker expression increases with age and LPS. (A–H) CD11b. (A–D) Hippocampus. A – 4 month old saline, B – 4 month old LPS, C – 21 month old saline, D – 21 month old LPS. (E–G) Cerebellum. E – 4 month old saline, F – 4 month old LPS, G – 21 month old saline, H – 21 month old LPS. (I–P) CD68. (I–L) Hippocampus. I – 4 month old saline, J – 4 month old LPS, K – 21 month old saline, L – 21 month old LPS. (M–P) Cerebellum. M – 4 month old saline, N – 4 month old LPS, O – 21 month old saline, P – 21 month old LPS.

**Fig. 2:**
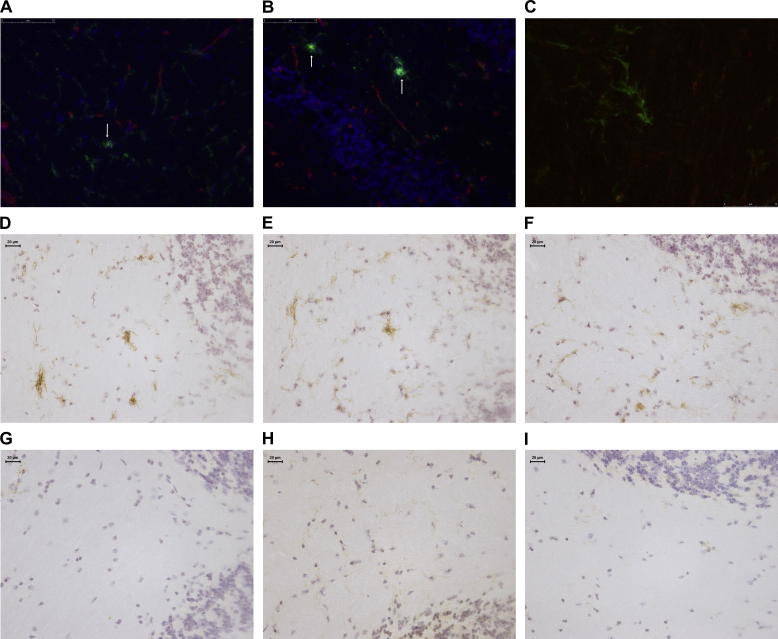
Microglial membrane marker expression increases with age. (A, B) Double immunofluorescence staining for CD11b (green) and collagen IV (red) in the fimbria (A) and cerebellar inferior peduncle (B) of a 21 month old mouse. CD11b positive cell aggregates are indicated by white arrows. The aggregates contain multiple nuclei and are not situated directly adjacent to blood vessels, as indicated by collagen IV positive basement membranes. Nuclei are blue after DAPI staining. (C) Double immunofluorescence staining for CD11c (green) and Ki67 (red), a marker of proliferation, in the cerebellar inferior peduncle. CD11c positive aggregates do not co-localise with Ki67 positive nuclei. D&G – CD11c staining in the cerebellar inferior peduncle of a 21 month old (D) and 4 month old (G) mouse. E&H – FcγRI staining in the cerebellar inferior peduncle of a 21 month old (E) and 4 month old (H) mouse. F&I – F4/80 staining in the cerebellar inferior peduncle of a 21 month old (F) and 4 month old (I) mouse.

Many, but not all, microglia exhibited a change in morphology in the aged brain (Figs. 1 and 2), including a thickening and de-ramification of processes and hypertrophy of the cell body (Fig. 1C and G). Morphological changes were observed in all regions studied, and microglia broadly retained the pattern that has previously been reported in grey versus white matter (Lawson et al., 1990), with longitudinal processes that run parallel to the axonal tracts in the white matter and radially branched microglia in the grey matter. Aged mice exhibited cell aggregates of approximately 20–30 μm in diameter, containing multiple nuclei and fewer, shorter, highly thickened processes (Fig. 1G, H, P). Some aggregates contained as many as 6 or 7 nuclei. These aggregates were predominantly found in the white matter, particularly in the cerebellum (Fig. 1G, H, P). Our results further show that systemic LPS challenge did not appear to change the morphology of the microglia or the number of multinucleate aggregates observed in aged mice (Fig. 1).

### 3.2 Microglial phenotype changes with age

In addition to morphological changes we noted distinct phenotypic changes in the aged brain, including increased expression of CD11b (Fig. 1A–H), CD68 (Fig. 1I–P), CD11c (Fig. 2D and G), FcγRI (Fig. 2E and H) and F4/80 (Fig. 2F and I). The phenotype changes were more pronounced in the cerebellum compared to the hippocampus. Increased levels of CD68 were found on microglia in the white matter regions of the cerebellum of aged brain, while in the young brain immunoreactivity for this marker was predominantly associated with perivascular macrophages (Fig. 1M and O). Double immunofluorescence showed that cell aggregates in the aged brain are microglia as CD11b positive aggregates were not associated with blood vessels and mainly found in the parenchyma, and are therefore not components of the perivascular macrophage population (Fig. 2A and B). Some aggregates extended processes that made contact with vasculature, but most did not. We also show that these aggregates were not groups of proliferating cells by double staining for CD11c and Ki67 (Fig. 2C). Expression of CD11c, FcγRI and F4/80 was very weak or not detectable in the 4 month old brain (Fig. 2G–I), but all three markers were robustly expressed in aged cerebellar white matter (Fig. 2D–F). In summary, age dependent changes in morphology and phenotype appear to arise in a region dependent manner, with a specific white matter phenotype present in the aged brain, in particular in the cerebellum.

### 3.3 Quantification of microglial phenotype changes with age: CD11b, CD68 and F4/80

**Fig. 3:**
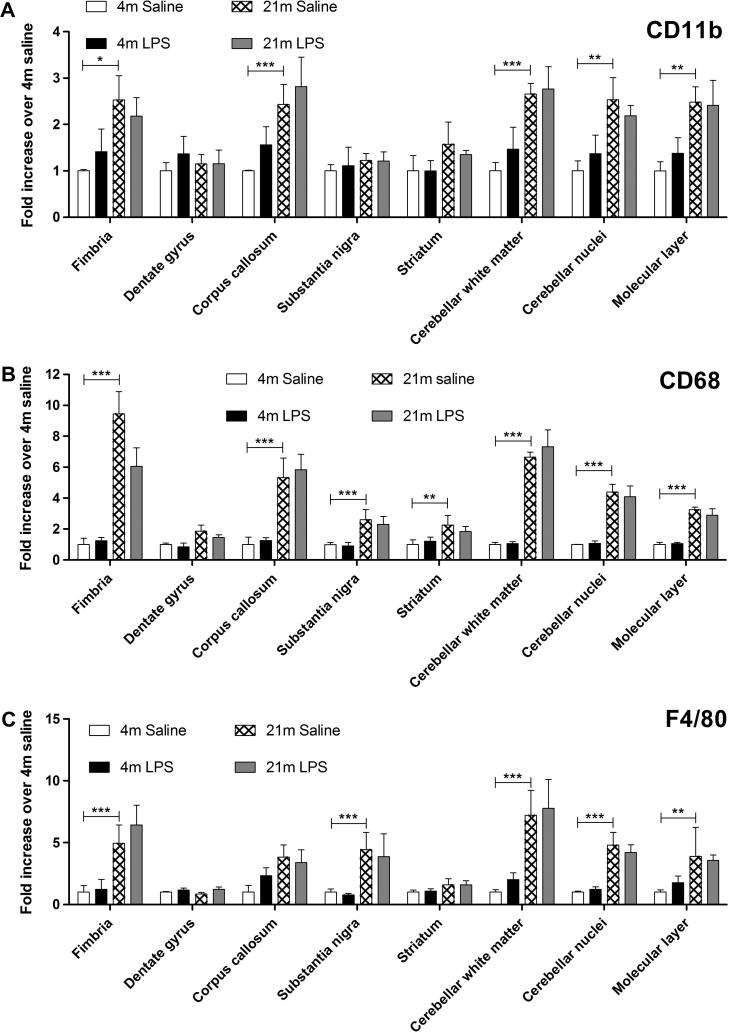
Age related changes in microglial phenotype depends on brain region. Tissue collected and analysed 24 h after intraperitoneal injection of saline or LPS. (A) CD11b expression in selected regions of the CNS. CD11b expression was significantly influenced by age, region and age × region (*p* < 0.05), but not LPS. *n* = 4–5 per region. (B) CD68 expression in selected regions of the CNS. CD68 expression was significantly influenced by age, region and age × region, but not LPS (*p* < 0.001). *n* = 4–5 per region. (C) F4/80 expression in selected regions of the CNS. F4/80 expression was significantly influenced by age, region and age × region (*p* < 0.001), but not LPS. *n* = 4–5 per region. Horizontal bars denote a significant effect of age (4 m saline to 21 m saline) or LPS (21 m saline to 21 m LPS) within that region. Data is shown as mean ± SEM. ^∗^ denotes *p* < 0.05; ^∗∗^ denotes *p* < 0.01; ^∗∗∗^ denotes *p* < 0.001.

We quantified the expression levels of functional markers in the different regions studied. In the ageing brain an increased expression of CD11b, CD68 and F4/80 (Fig. 3, *n* = 5 per group): for all three markers there was a strong effect of age on expression level (CD11b: *F*_(1,111)_ = 38.35, *p* < 0.001; CD68: *F*_(1,108)_ = 271.36, *p* < 0.001; F4/80: *F*_(1,109)_ = 75.86, *p* < 0.001). None of these markers were significantly affected by systemic LPS 24 h after injection. Region had a strong effect on expression of all three markers, (CD11b: *F*_(7,111)_ = 2.45, *p* = 0.022; CD68: *F*_(7,108)_ = 7.90, *p* < 0.001; F4/80: *F*_(7,109)_ = 4.64, *p* < 0.001). We detected an interaction between age and region for expression of all three markers (CD11b: *F*_(7,111)_ = 2.12, *p* = 0.047; CD68: *F*_(7,108)_ = 7.789, *p* < 0.001; F4/80: *F*_(7,109)_ = 4.64, *p* < 0.001), suggesting that microglial activation is differentially affected by age in different brain regions. The increases in expression of CD11b, CD68 and F4/80 were greatest in the cerebellum and in particular in the cerebellar inferior peduncles. Microglial expression of all three markers in the fimbria and for CD11b and CD68 the corpus callosum was also strongly increased in aged animals (Fig. 3A and B). Changes in the expression of these molecules in the white matter were greater than those in the grey matter. The dentate gyrus did not exhibit any changes in expression with ageing for any of these three markers.

### 3.4 Quantification of microglial phenotype changes with age: CD11c and FcγRI

**Fig. 4:**
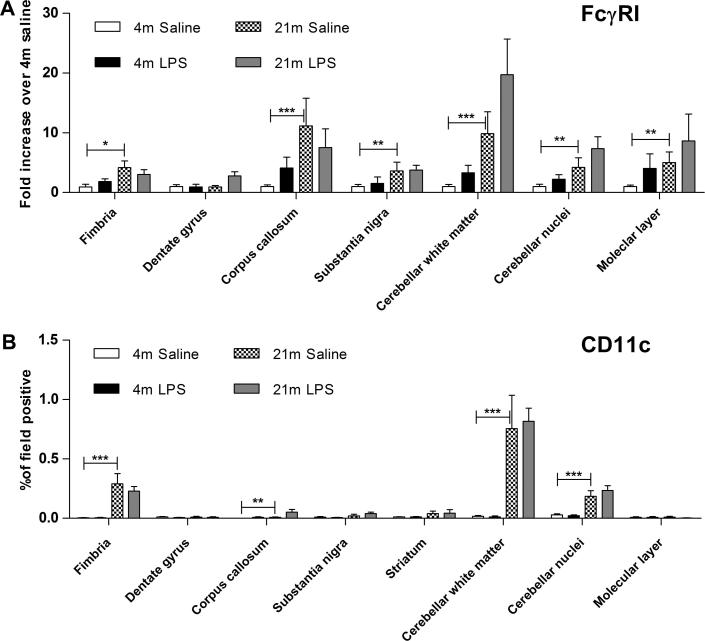
Age related changes in CD11c and FcγRI expression depends on brain region. Tissue collected and analysed 24 h after intraperitoneal injection of saline or LPS. (A) CD11c expression in selected regions of the CNS. CD11c expression was significantly influenced by age, region and age × region (*p* < 0.001), but not LPS. *n* = 5 per region. (B) FcγRI expression in selected regions of the CNS. FcγRI expression was significantly influenced by region, age and LPS (*p* < 0.05). *n* = 4–5 mice per region. Horizontal bars denote a significant effect of age (4 m saline to 21 m saline) or LPS (21 m saline to 21 m LPS) within that region. Data is shown as mean ± SEM. ^∗^ denotes *p* < 0.05; ^∗∗^ denotes *p* < 0.01; ^∗∗∗^ denotes *p* < 0.001.

The expression levels of CD11c (Fig. 4A) and FcγRI (Fig. 4B) were also quantified and expression of both was significantly increased by age (CD11c: *F*_(1,128)_ = 63.08, *p* < 0.001; FcγRI: *F*_(1,92)_ = 61.37, *p* < 0.001), region (CD11c: F_(7,128)_ = 15.76, *p* < 0.001; FcγRI: *F*_(6,92)_ = 4.84, *p* < 0.001) and, for FcγRI, LPS injection (*F*_(1,92)_ = 5.97, *p* < 0.05). An interaction between age and region was detected for CD11c expression (*F*_(7,128)_ = 11.72, *p* < 0.001), but not FcγRI. Strikingly, CD11c expression was up-regulated exclusively in white matter regions during ageing. All four white matter regions examined demonstrated a significant increase in CD11c expression with age (Fig. 4A) and the most caudal area of white matter studied, the inferior cerebellar peduncle, exhibited the greatest increase in expression, but CD11c expression was not further influenced by systemic LPS. Although expression of FcγRI was increased in all regions of the ageing brain, changes in FcγRI expression were more pronounced in white matter areas and the cerebellum than in the hippocampus of 21 month old mice (Fig. 4B). FcγRI expression after LPS injection was also highest in the three cerebellar regions investigated.

Changes in other molecules expressed by microglia during ageing and after systemic LPS injection were investigated in a qualitative manner using immunohistochemistry (data not shown). A small number of Dectin-1 positive cells were detected in the white matter tracts of aged animals (3–4 cells per ×20 field of cerebellum), but not in aged grey matter or young white matter. The expression levels of Dectin-1 were not influenced by systemic LPS. DEC-205 positive cells were not observed in either the young or aged brain. We also investigated FcγRII/III and MHCII expression levels and the majority of positive cells were associated with blood vessels. We could not detect any noticeable changes in the expression of these two molecules on microglia dependent on age or LPS. In summary, age related changes in expression of microglia associated molecules varied greatly between different brain regions, with the cerebellum and the white matter showing the most pronounced changes, while the effect of systemic LPS on microglia associated molecule expression was limited to FcγRI.

### 3.5 Aged animals show an exaggerated decline in burrowing in response to systemic LPS

**Supplementary Fig. 1:**
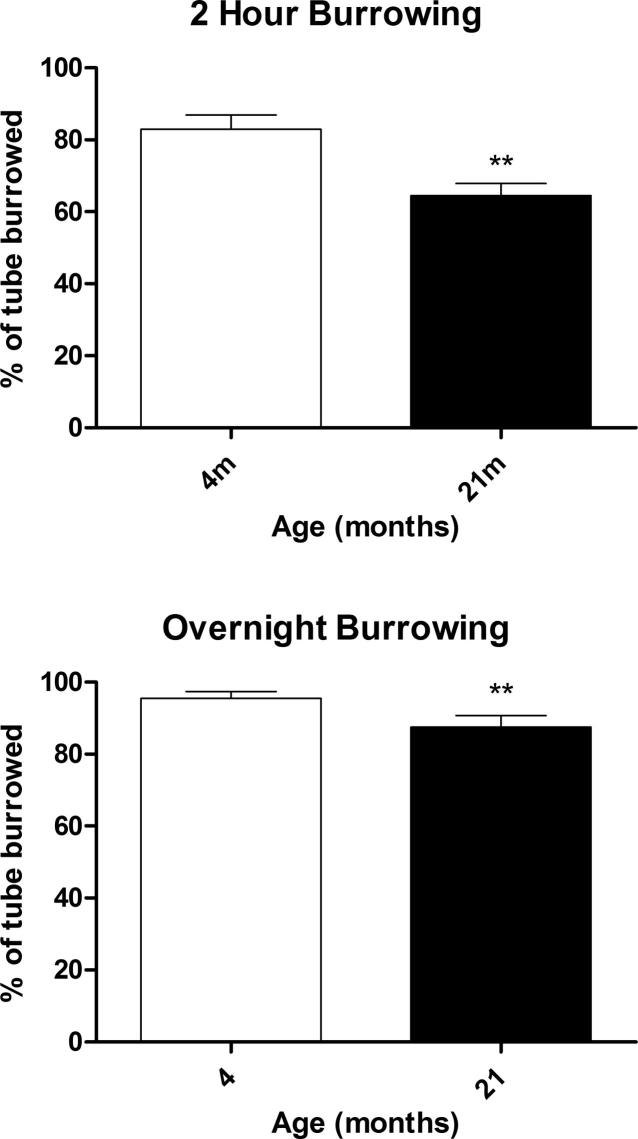


**Fig. 5:**
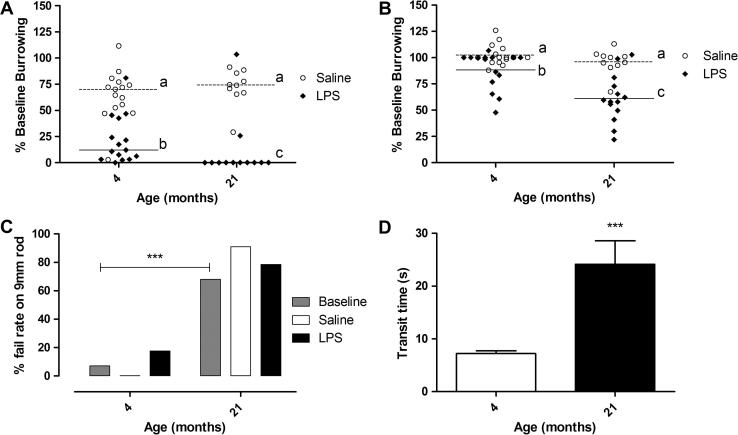
Behavioural changes in young and aged mice and the effect of systemic inflammation. (A) Burrowing measured between 3 and 5 h after intraperitoneal injection of LPS (100 μg/kg) or saline. Data shows systemic LPS induced a greater decline in 21 month old mice compared to 4 month old mice. Horizontal bars represent medians. (B) Overnight burrowing after intraperitoneal injection of LPS or saline. Data shows that recovery of burrowing is slower in 21 month old mice compared to 4 month old mice. Horizontal bars represent means. (C) Percentage fail rate in the 9 mm static rod test measured at baseline or between 1 and 2 h after intraperitoneal LPS or saline injection. 21 month old mice failed more frequently in the 9 mm static rod test than 4 months old. This change in performance was independent of strength (Supplementary Fig. 2). Pass/fail ratios were not influenced by injection of saline (open bars) or LPS (black, filled bars). Columns represent the percentage of mice which fail the task. As this graph is representation of binomial data drawn from a single experiment, no error bars are presented. (D) 9 mm static rod transit time to reach platform at baseline. Transit times reflect a decline in performance with age. Data is shown as mean ± SEM. ^∗∗∗^*p* < 0.001. Letters denote a significant difference between groups (*p* < 0.05).

To investigate whether the age related, region specific changes in microglial phenotype were associated with compromised CNS function, we performed behavioural assays dependent on two of the regions analysed for phenotype changes – the hippocampus and the cerebellum. We used burrowing as a measure of hippocampus dependent sickness behaviours (Deacon et al., 2002). A small decline in burrowing activity was seen at baseline with age, which may be attributable to changes in baseline locomotor activity (Supplementary Fig. 1). Between 3 and 5 h after a systemic LPS injection all mice showed a decline in burrowing, with a greater decline in activity in aged mice compared to young mice (Fig. 5A) (LPS group: *p* < 0.001, *n* = 14–15). Most 21 month old mice failed to show any burrowing activity (median = 0%), whereas the majority of 4 month old mice retained a degree of burrowing activity (median = 12.1%). There was no age-related effect of saline injection on burrowing (*p* = 0.233, *n* = 10–15). At 24 h after injection the LPS-challenged mice had partially recovered their burrowing activity (Fig. 5B), but this recovery was significantly less in the 21 month old mice compared to the 4 month old mice (4 vs 21 month group *p* < 0.001. *n* = 14–15). Age and LPS both had a significant effect on overnight burrowing (Age: F_1,__50_ = 13.34, *p* < 0.001. LPS: F_1,__50_ = 28.21, *p* < 0.0001). In addition, an interaction between the two factors was detectable (F_1,__50_ = 5.053, *p* = 0.029). To conclude, a systemic challenge of LPS led to an exacerbated and decrease in burrowing activity in 21 month old mice when compared to 4 month old mice.

### 3.6 Static rod performance declines with age, but is not modulated by systemic LPS

**Supplementary Fig. 2:**
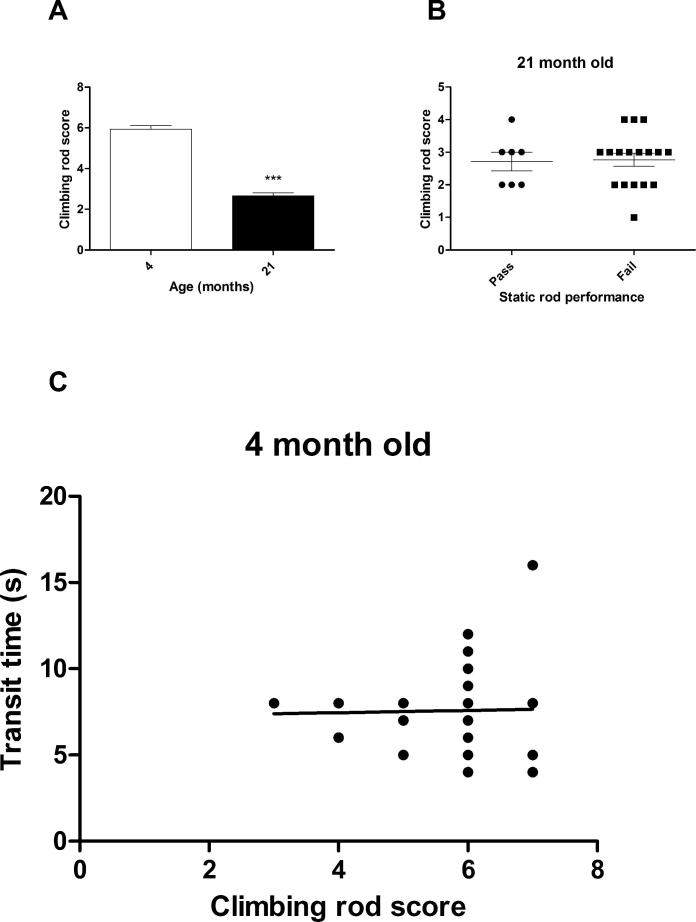


Next, we investigated a cerebellum dependent behaviour, the multiple static rod test, which assesses the co-ordination and balance of mice on different diameter static rods (Carter et al., 1999; Contet et al., 2001). Mice were placed on a suspended 9 mm diameter static rod and the transit time to reach a platform after orientation was assessed in saline and LPS-treated mice (Fig. 5C and D). Chi squared analysis of baseline static rod performance showed a significant difference between young (7%, *n* = 30) and aged (68%, *n* = 25) mice in pass/fail ratios on the 9 mm static rod (х^2^ = 22.69, d.f. = 1, *p* < 0.0001) (Fig. 5C). Analysis of baseline transit times also showed a significant difference between young and aged mice (Mann Whitney test, *p* < 0.0001, *n* = 25–30 per group) (Fig. 5D). Injection of LPS or saline did not have a significant effect on pass/fail rates at any age and there were not sufficient successful completions of the test in the 21 month old mice to test for differences in transit times after injection. We also tested muscle strength using the climbing rod test to investigate whether changes in muscle strength correlated with poorer static rod performance. There was a decline in climbing rod performance with age (*p* < 0.0001, Mann Whitney test; supplementary data Fig. 2A), but we found no difference in climbing rod performance between 21 month old mice that passed or failed the static rod test (supplementary data Fig. 2B). There was also no correlation between climbing rod test performance and static rod transit time in 4 month old mice (Supplementary data Fig. 2C).

Finally we investigated the effect of LPS injection on the expression of inflammatory mediators in the different CNS regions of aged and young mice using quantitative real time PCR. However, we could not detect any significant increase of IL-6, IL-1β or iNOS mRNA expression 24 h after LPS injection in young or aged cerebellum or hippocampus (data not shown).

## 4 Discussion

In this study we have investigated the phenotype and morphological changes of microglia in eight distinct regions of the young and aged mouse brain. We show that age-related phenotype changes of microglial cells are more pronounced in the white matter, with the cerebellum, the most caudal structure studied, showing the greatest differences.

Variations in microglial density have been well described in adult mouse brain with the hippocampus and substantia nigra exhibiting the highest and the cerebellar cortex the lowest density of microglia (Lawson et al., 1990). A number of studies in several areas of the CNS indicate that there is little, if any, change in the distribution or numbers of microglia with age (Deng et al., 2006; Long et al., 1998; Ogura et al., 1994; Peters et al., 2010), but less is known about phenotype changes in different regions of the aged mouse brain. Our results are in accord with a recent study describing regional variation in expression levels of immunoregulatory molecules in the healthy adult mouse brain. De Haas et al. showed that regional differences between microglial phenotypes in the adult mouse brain are subtle: expression levels of surface markers such as CD11b, CD40 and the fractalkine receptor CX3CR1 appeared higher in the microglia of the spinal cord and cerebellum than the hippocampus (De Haas et al., 2008). In our study all functional markers tested displayed the greatest increase in expression with age in white matter regions, particularly in the cerebellum, identifying a clear trend in phenotype changes along the rostro-caudal axis in the aged mouse brain.

### 4.1 Microglial phenotype changes in response to injury along the rostro-caudal axis

Phenotype changes in microglia are well described in response to acute and chronic injury or disease, but only a few studies have looked at differential responsiveness to the grey matter versus the white matter along the rostro-caudal neuraxis. Trauma-induced lesions lead to a greater microglial response in the spinal cord than the cortex or corpus callosum and the spinal white matter exhibited a greater microgliosis than spinal grey matter (Batchelor et al., 2008; Schnell et al., 1999a). Regional differences in responsiveness to inflammatory stimuli are partly responsible for these observations, as stereotaxic injections of recombinant cytokines into the striatum fail to evoke a robust response, while similar injections into the spinal cord or brainstem are associated with BBB breakdown, microgliosis and secondary tissue damage (Campbell et al., 2002; Phillips and Lampson, 1999; Phillips et al., 1999; Schnell et al., 1999b). This regional difference in responsiveness to inflammatory stimuli is also evident in EAE, which targets the spinal cord rather than more rostral regions of the brain, such as the forebrain (Sun et al., 2004). Collectively, these studies suggest that the caudal and white matter regions of the CNS are more responsive and therefore more vulnerable to inflammatory stimuli. Our study suggests that the differential sensitivity of these microglial populations also applies to the ageing process. We show that in the aged brain there is a greater up-regulation of CD11b, CD11c, CD68, F4/80 and FcγRI in white matter than in grey matter and more in caudal areas than rostral areas. These data are in agreement with previous studies in the aged rat brain suggesting a rostral caudal gradient of microglial activation (Kullberg et al., 2001).

### 4.2 Pronounced white matter related changes

It has been previously reported that the microglia of the white matter express greater levels of microglia associated molecules with age than those of the grey matter (Kullberg et al., 2001; Perry et al., 1993), and that the microglia may appear in “clumps” of immunoreactive membranes in white matter (Perry et al., 1993; Stichel and Luebbert, 2007). Our study shows that these aggregates are not directly associated with blood vessels and are not clusters of proliferating cells. Macrophages and microglia are known to form multinucleate giant cells through fusion under a variety of inflammatory conditions (Fendrick et al., 2007; Gasser and Most, 1999; Suzumura et al., 1999). Whether these cells are aggregates of individual microglia or a single syncytium is not clear from our study, but the appearance of multinucleate giant cells during ageing would represent a significant alteration in microglial phenotype and function.

### 4.3 Pronounced changes in CD11c expression

We observed a significant increase in CD11c expression levels, predominantly in the white matter of the cerebellum. CD11c is a protein found at high levels on dendritic cells, but is also found on macrophages and microglia under neuroinflammatory conditions (Reichmann et al., 2002; Remington et al., 2007). Increases in CD11c immunoreactivity with age have been reported previously with robust CD11c expression in the aged white matter and occasional CD11c expression throughout the grey matter (Kaunzner et al., 2010; Stichel and Luebbert, 2007). These studies describe CD11c positive cells as dendritic cells, as they express DEC-205, MIDC8, MHCII and the co-stimulatory molecules CD80 and CD86. Using immunohistochemistry we did not detect DEC-205 or MHCII expression in the aged brain. This discrepancy may be explained by the superior sensitivity of alternative methods of detection, such as flow cytometry, or by the strain of mice used (Henry et al., 2009). The functional consequence and mechanism underlying increased expression of CD11c in the aged brain is unknown, but increased turnover of myelin with age may be a contributing factor (Ando et al., 2003). It has been shown that engagement of low density lipoprotein receptor 1 (LRP-1) on macrophages results in increased expression of CD11c (Cho et al., 2007; Gower et al., 2011). Ligands for LRP-1 include low density lipoprotein (Kuchenhoff et al., 1997) and the myelin component MBP-1 (Gaultier et al., 2009). Whether the CD11c + cells in our study are microglia that have taken up myelin components/breakdown products, or infiltrating dendritic cells or macrophages remains to be determined.

### 4.4 Neurodegeneration in the ageing brain

It is well recognised that the microglia are exquisitely sensitive to neurodegeneration. However, in rodents and primates the extent to which neurodegeneration occurs in the ageing CNS varies considerably from region to region. The substantia nigra (Ma et al., 1999; Mouton et al., 2010) and cerebellum (Sturrock, 1989; Woodruff-Pak et al., 2010) exhibit significantly greater age-related neuronal loss than the hippocampus (Calhoun et al., 1998; Rapp and Gallagher, 1996) or striatum (Pesce and Reale, 1987), and substantial loss of myelinated axons has been reported in white matter regions (Bowley et al., 2010; Cavallotti et al., 2001; Sandell and Peters, 2002, 2003). The areas with greatest neuronal loss are also the regions that exhibit greater changes in microglial phenotype.

Whether neuronal loss drives microglial phenotype changes in ageing, or if changes to the microglia precede and contribute towards neuronal loss, is not known. There are however several mechanisms by which neurons and oligodendrocytes keep microglia in a quiescent state, such as interactions between CD200, fractalkine or CD47 and their cognate receptors on microglia (Gitik et al., 2011; Hoek et al., 2000; Kong et al., 2007; Koning et al., 2009; Lyons et al., 2009). Two studies in the healthy adult mouse brain have revealed significant regional variations in the distribution of these molecules. Koning et al. (2009) observed that CD200 expression is greater in grey than white matter, which may contribute to the regional differences in microglial phenotype we report in this study. Fractalkine transcript expression has been reported to be significantly lower in the cerebellum and other caudal areas such as the brainstem than the hippocampus or striatum, which may help to explain the rostral caudal gradient of microglial phenotype changes (Tarozzo et al., 2003). Decreased expression of CD200 in the hippocampus and substantia nigra (Frank et al., 2006; Wang et al., 2011), and of fractalkine in the hippocampus and forebrain have been demonstrated in aged mice (Lyons et al., 2009; Wynne et al., 2010). Increased numbers of multinuclear giant cells have also been observed in CD200-/- mice (Hoek et al., 2000), providing a possible explanation for their presence in the aged brains of our study. A wider assessment of the expression of these immunoregulatory molecules in different regions of the aged brain and how they may correlate with changes in microglial phenotype would be of interest.

### 4.5 Impact of systemic inflammation on aged-related microglia phenotype

We anticipated an increase in expression levels of microglia associated molecules after systemic LPS injection, which has previously been shown to up-regulate FcγRI (Lunnon et al., 2011) and CD11b (Buttini et al., 1996). However, the only molecule we found to be sensitive to systemic LPS injection was FcγRI. CD11b expression was not significantly altered 24 h after systemic LPS challenge. Furthermore, the effect of systemic LPS on FcγRI expression was subtle, region dependent and primarily observed in the white matter regions and the cerebellum of both young and aged mice. A later time point post injection, such as three days, may yield a more robust effect on expression of these molecules (Buttini et al., 1996).

### 4.6 Age-related functional deficits

Since we had shown that the molecular expression patterns of the microglia in distinct CNS regions were altered with age we used behavioural assays to assess the functional integrity of two regions, the hippocampus and the cerebellum. We demonstrate a significant age-related deficit in static rod test performance and verified that this was not dependent on loss of muscle strength. There is a progressive loss of Purkinje neurons with age (Woodruff-Pak et al., 2010) and Purkinje neuron specific degeneration has previously been shown to compromise the performance of mice in tasks assessing co-ordination and balance (Chen et al., 1996; Kyuhou et al., 2006). A correlation of conditioned eye blink response with Purkinje neuron numbers has also been previously shown, suggesting that Purkinje cell loss may be the critical component of age-related cerebellar dysfunction (Woodruff-Pak, 2006). LPS injection did not exacerbate deficits in performance in this task at any age, suggesting the cerebellar circuitry controlling static rod performance is not sensitive to systemic LPS.

Burrowing is a hippocampus dependent (Deacon et al., 2002), species typical behaviour that is sensitive to systemic inflammatory challenge (Teeling et al., 2007). We demonstrated that aged mice exhibit an exaggerated response and a delayed recovery from systemic LPS challenge. Exaggerated sickness behaviour in aged animals in response to systemic inflammatory challenge has been previously reported (Barrientos et al., 2006; Godbout et al., 2005, 2008; McLinden et al., 2011), but this is the first study to use burrowing in response to systemic LPS treatment in an ageing context. Elevated levels of cytokines within the aged hippocampus have been demonstrated following systemic inflammatory challenge (Barrientos et al., 2009; Chen et al., 2008; Godbout et al., 2005), which are likely produced by primed microglia in the aging brain (Frank et al., 2010; Wynne et al., 2010). We were not able to demonstrate the presence of inflammatory cytokines or iNOS 24 h after systemic LPS injection in any brain region studied. We had anticipated that elevation of these molecules would be prolonged in aged animals in line with other studies (Godbout et al., 2005; Wynne et al., 2010). This discrepancy may be due to our use of a lower dose of LPS (100 μg/kg vs 330 μg/kg) and a different sex and strain of mouse (male BALB/c vs female C57/BL6). Our data does not however exclude the possibility of an exaggerated local inflammatory at an earlier time-point following systemic LPS injection.

## 5 Summary

In this study we have demonstrated significant differences in microglial phenotypes between distinct regions of the aged brain. The microglia of the white matter show more robust changes than those of grey matter and there is evidence of a rostro-caudal gradient in the magnitude of these changes. The age-related changes in microglia phenotype reported here may be of particular interest when comparing studies in rodent and human material. In humans white matter makes up ∼40% of the adult human brain (Gur et al., 1999) compared to 10% in the mouse (Zhang and Sejnowski, 2000), and human white matter contains a greater density of microglia than grey matter (Mittelbronn et al., 2001), conversely to the mouse (Lawson et al., 1990). The functional significance of these grey/white matter differences in microglial phenotype during ageing remain to be elucidated.

## Conflict of interest statement

All authors declare that there are no conflicts of interest.

## Figures and Tables

**Table 1:** List of primary antibodies used.

Antibody target	Clone	Animal of origin	Source
CD11b	5C6	Rat-anti-mouse	Serotec (Oxford, UK)
CD11c	N148	Hamster-anti-mouse	Kind gift from Prof. M. Glennie (University of Southampton)
CD68	FA-11	Rat-anti-mouse	Serotec (Oxford, UK)
F4/80	CI:A-1	Rat-anti-mouse	Serotec (Oxford, UK)
FcγRI	290322	Rat-anti-mouse	R&D Systems (Abingdon, UK)
MHCII	M5/114.15.2	Rat-anti-mouse	Abcam (Cambridge, UK)
CD16/32	FCR4G8	Rat-anti-mouse	Serotec (Oxford, UK)
Dectin-1	218820	Rat-anti-mouse	R&D Systems (Abingdon, UK)
Ki67	Polyclonal	Rabbit-anti-mouse	Abcam (Cambridge, UK)
DEC-205	NLDC-145	Rat-anti-mouse	Serotec (Oxford, UK)
Collagen IV	Polyclonal	Rabbit-anti-mouse	Serotec (Oxford, UK)
